# Physical and motivational effects of Exergames in healthy adults—Protocol for a systematic review and meta-analysis

**DOI:** 10.1371/journal.pone.0266913

**Published:** 2022-04-19

**Authors:** Katrin Hoffmann, Josef Wiemeyer

**Affiliations:** Institute of Sport Science, Technical University Darmstadt, Darmstadt, Hessen, Germany; Universiti Sains Malaysia, MALAYSIA

## Abstract

Exergames have the potential to be used as motivating physical training tools. Numerous studies are currently available that investigate the effects of physical training with Exergames. However, most studies focus on specific training effects or specific target groups. A comprehensive summary of conditioning, coordinative and sports skill related training effects with Exergames in healthy adults is still missing. This contribution presents the protocol for a systematic review that aims to: a) summarize absolute and relative effects of training with Exergames on physical indicators and motivation compared to no or conventional training; b) identify possible mediators and moderators for varying training responses; c) identify gaps in the current evidence related to Exergame-based training.

## Introduction

Exergames or Active Video Games (AVG) are digital games, that require the player to engage in physical activity including upper or lower body movement to control the game [1]. Since Exergames have the potential to be used as motivating physical training tools, they have gained increasing attention in the last decades. Up to date, numerous studies investigating Exergames are available that focus on specific training effects or specific target groups.

However, a recent overview of reviews revealed that a comprehensive summary of training effects in terms of conditioning, coordination and sports skills is still missing [2]. Current reviews are either focused on specific samples (e.g. children and adolescents, older adults with or without clinical issues) or on selected aspects of physical exercise. In particular, reviews focusing on training effects regarding balance, strength, or motor skill training in healthy adults were not found. Therefore, a lack of reviews addressing the broad effects of training with Exergames or AVG in healthy adults was identified.

This paper introduces the protocol for a systematic review [3] aiming to: a) summarize absolute and relative effects of training with Exergames on physical indicators and motivation compared to no or conventional training; b) identify possible mediators and moderators for varying training responses; c) identify gaps in the current evidence related to Exergame-based training.

## Material and methods

This systematic review will be registered in PROSPERO.

The review protocol was developed following Cochrane`s guidelines of reviews [4] and the recommendations in PRISMA-P for Protocols [5] (S1 Checklist). Research question for this review was developed using PICOS (Population, Interventions, Comparators, Outcomes, Study design) elements.

## Eligibility criteria

The following paragraph describes the inclusion and exclusion criteria for the integration of studies.

### Types of studies

Only randomized trials will be considered for inclusion. No restriction regarding the type or styles of randomized trials will be applied in order to ensure an inclusion of all relevant literature. Theoretical studies that use text and opinions as their primary source of evidence as well as case and qualitative studies will be excluded [6].

### Types of participants

All interventions targeting healthy adults will be included. This comprises the age from 18 to 64 years according to the recommendations of the World Health Organization [7]. No restriction regarding training status (e.g., trained, sedentary) will be applied. Reviews targeting treatments in participants with diseases (e.g. cardiovascular, neural or mental disorders) will be excluded. No restrictions regarding gender, ethnicity, geographic location, specific racial or cultural interests will be applied.

### Types of interventions

Only treatments including training interventions performed with Exergames or AVG will be considered. Training interventions with Exergames supplemented by additional components will only be considered if these additional components did not exert conflicting or confounding influence (e.g., knowledge transfer). Due to possible monitoring issues, home-based training will be excluded.

### Types of comparators

Only interventions with control groups that performed either conventional physical training (active control; relative effects) or no training (passive control; absolute effects) will be included.

### Types of outcome

Effects regarding endurance or aerobic capacity, strength or resistance, flexibility or agility, speed, balance, complex motor reactions or sensorimotor coordination, sports skills [8, 9] will be considered as primary outcomes. Furthermore, player experience [10, 11] representing motivation during gameplay will be considered as primary outcome. These outcomes are planned to be assessed as pre-post differences comparing baseline to post-intervention performance. Adverse events, physical activity level, attitudes, and knowledge [10] will be considered as secondary outcomes.

### Types of measures

Only reliable and valid measures of physical training effects and player experience effects will be considered [9, 12]. These measures comprise physiological and biomechanical parameters as well as testing routines and questionnaires. Examples of valid indicators of physical training effects include but are not limited to: heart rate, oxygen uptake or lactate level at distinct stress levels or maximum oxygen uptake (endurance); power output or jump tests (e.g., vertical jump test—strength), range of motion (flexibility), sprint tests (e.g., 100m sprint—speed), body’s center of gravity tests (balance), sensorimotor coordination tests (complex motor reactions), and sports-specific skill tests (sport skills). Player experience is assessed by questionnaires (e.g., game experience questionnaire (GEQ [13]) or physical activity enjoyment scale (PACES [14]—motivation).

### Search strategy

An electronic search will be performed in Science Research, EBSCOhost, the Web of Science Core Collection, and SURF. For Science Research, the advanced search comprises the topics “Computer & Technology”, “Health & Medicine”, and “Multidisciplinary Sources”. The EBSCOhost platform includes the following databases: INSPEC, Library, Information Sciences & Technology Abstracts, “APA PsycArticles, APAPsycInfo, SPORTDiscus with Full Text, and MEDLINE.

All databases will be searched using the keywords presented in Table 1 in all fields. Both groups of keywords will be combined with the Boolean operator “AND”.

**Table 1:** Groups and function of used keywords.

Keywords	Function
Exergam* OR Active Video Gam*	Specifies the field
endurance OR aerobic* OR stamina OR strength OR resistance OR flexibility OR agility OR speed OR balance OR complex motor reaction* OR sensorimotor coordination OR sport skill* OR motivation	Specifies the scope relevant to the research question

In addition, a manual search analyzing the reference lists of all kinds of reviews will be performed to identify studies that were not found by the electronic research. The search results will also be presented to further experts in the field of Exergames to identify additional literature.

Only studies published under peer-review conditions will be considered without restriction regarding year of publication. In order to enable a check of language bias, no restriction regarding the language of publication will be applied in the first search phase. In the further review process, only literature published in German or English will be considered. Language bias will be checked by comparing the search results (German versus English versus other languages).

### Data analysis and extraction

The reference system CITAVI will be used for data management and analysis.

After removing duplicates, two reviewers will independently analyze the identified literature. Search results will first be evaluated by screening title and abstract. Full texts will then be evaluated to determine the studies meeting the defined inclusion criteria. The screening and selection process will be documented and reported using the PRISMA flow diagram [5].

The data extraction of eligible studies will be carried out using a data extraction sheet (S1 Data). This sheet analyzes:

- key information following the PICOS criteria (participants, interventions, comparisons, outcomes, study design)
- methodological quality of study (PEDro scale)) [15]
- quality of evidence of study (GRADE) [16]
- applied training normatives (frequency, intensity, type, time, volume, and progression–FITT-VP) [17].

In case of missing data, the original study authors will be asked to provide full reports of their methods and results. Studies will only be included if either full texts are available or all information is provided by the authors.

Any disagreement during the selection process will be resolved by discussion or by a third reviewer performing a consensus analysis.

### Data syntheses and statistical considerations

In case of sufficient number of available data a meta-analysis will be performed. The inter-rater reliability for the total scores of the methodological quality will be checked to analyze the level of agreement between the reviewers. The extracted data will be categorized according to PICOS. Heterogeneity between studies will be assessed using the I^2^ statistic. Significant inconsistency will be analyzed on a case-by-case basis. If necessary, subgroup and moderator analysis will be performed stratified by sex, applied Exergames, and training experience of the participants. Graphical analyses will be performed using funnel plots and forest plots.

## Dissemination plan

The intended deadline for completing the review is March 2022. The final review will be published in a peer-reviewed journal. All data will be available as supporting information. Furthermore, brief findings will be presented in academic meetings and relevant professional assemblies.

## Supporting information

S1 Checklist PRISMA-P-statement. (DOCX)

S1 Data Data extraction sheet. (XLSX)

## References

[1] Staiano AE. Exergames, Energy Expenditure, and Obesity. The International Encyclopedia of Media Psychology, 2021; 1–7.

[2] Hoffmann K, Wiemeyer J. Physical and motivational effects of Exergames in healthy adults: Overview of Reviews. In: Advances in Intelligent Systems and Computing: Proceedings of the 9^th^ International Performance Analysis Workshop and Conference and 5^th^ IACSS Conference. 2021. Forthcoming.

[3] Pollock M, Fernandes RM, Becker LA, Pieper D, Hartling L. Chapter V: Overviews of Reviews. In: Higgins JPT, Thomas J, Chandler J, Cumpston M, Li T, Page MJ, Welch VA, editors. Cochrane Handbook for Systematic Reviews of Interventions version 6.2 (updated February 2021). [Cited 2021 July]. Available from: www.training.cochrane.org/handbook(2021).

[4] Higgins JPT, Thomas J, Chandler J, Cumpston M, Li T, Page MJ, et al. Cochrane Handbook for Systematic Reviews of Interventions version 6.2 (updated February 2021). [Cited 2021 July]. Available from www.training.cochrane.org/handbook.

[5] Moher D, Liberati A, Tetzlaff J, Altman DG, The PRISMA Group. Preferred Reporting Items for Systematic Reviews and Meta-Analyses: The PRISMA Statement. PLoS Med. 2009; 6(7): e1000097. doi: 10.1371/journal.pmed.1000097 19621072 PMC2707599

[6] Aromataris E, Fernandez R, Godfrey C, Holly C, Khalil H, Tungpunkom P. Chapter 10: Umbrella Reviews. In: Aromataris E, Munn Z, editors. JBI Manual for Evidence Synthesis. 2020. pp. 360–405. doi: 10.46658/JBIMES-20-01

[7] Bull FC, Al-Ansari SS, Biddle S, Borodulin K, Buman MP, Cardon G, et al. World Health Organization 2020 guidelines on physical activity and sedentary behavior. British journal of sports medicine. 2020; 54(24): 1451–1462. doi: 10.1136/bjsports-2020-102955 33239350 PMC7719906

[8] Haff GG, Triplett NT. Essentials of strength training and conditioning. 4th ed. Champaign, Ill.: Human kinetics; 2015.

[9] Bös K. Handbuch motorische Tests: sportmotorische Tests, motorische Funktionstests, Fragebögen zur körperlich-sportlichen Aktivität und sportpsychologische Diagnoseverfahren. 3rd ed. Göttingen: Hogrefe Verlag; 2017.

[10] Caserman P, Hoffmann K, Müller P., Schaub M, Straßburg K, Wiemeyer J, et al. Quality criteria for serious games: serious part, game part, and balance. JMIR serious games. 2020; 8(3): e19037. doi: 10.2196/19037 32706669 PMC7414398

[11] Wiemeyer J, Nacke N, Moser C, Mueller F. Player experience. In: Dörner R, Göbel S, Effelsberg W, Wiemeyer J, editors. Serious Games—Foundations, Concepts and Practice. Cham: Springer International Publishing; 2016. pp. 243–271. doi: 10.1007/978-3-319-40612-1_9

[12] Atkinson G, Nevill AM. Statistical methods for assessing measurement error (reliability) in variables relevant to sports medicine. Sports medicine. 1998; 26(4): 217–238. doi: 10.2165/00007256-199826040-00002 9820922

[13] Nacke L. Affective Ludology: Scientific measurement of user experience in interactive entertainment. Doctoral Dissertation. Karlskrona, Schweden: Blekinge Insitute of Technology Doctoral Dissertation Series–Printfabriken.

[14] Kendzierski D, DeCarlo KJ. Physical activity enjoyment scale: two validation studies. Journal of Sport and Exercise Psychology. 1991; 13(1): 50–64.

[15] Maher CG, Sherrington C, Herbert RD, Moseley AM, Elkins M. Reliability of the PEDro scale for rating quality of randomized controlled trials. Physical therapy. 2003; 83(8): 713–721. 12882612

[16] Balshem H, Helfand M, Schünemann HJ, Oxman AD, Kunz R, Brozek J, et al. GRADE guidelines: 3. Rating the quality of evidence. Journal of clinical epidemiology. 2011; 64(4): 401–406. doi: 10.1016/j.jclinepi.2010.07.015 21208779

[17] American College of Sports Medicine. ACSM’s Guidelines for Exercise Testing and Prescription, 10th ed. Philadelphia (PA): Lippincott Williams & Wilkins; 2017.

